# Unwitting Accomplices: Endocrine Disruptors Confounding Clinical Care

**DOI:** 10.1210/clinem/dgaa358

**Published:** 2020-07-02

**Authors:** Matthew Genco, Lisa Anderson-Shaw, Robert M Sargis

**Affiliations:** 1 Department of Medicine, University of Illinois at Chicago, Chicago, IL, US; 2 Department of Medical Education, University of Illinois at Chicago, Chicago, IL, US; 3 Chicago Center for Health and Environment (CACHET); University of Illinois at Chicago, Chicago, IL, US

## Abstract

Burgeoning evidence over the last 25 years has identified myriad synthetic chemicals with the capacity to alter various aspects of hormone synthesis and action. These endocrine-disrupting chemicals (EDCs) have been linked to various diseases, including reproductive disorders, metabolic diseases, and developmental abnormalities, among others. Exposure to EDCs arises from industrial activity, use of personal and home care products, and consumption of contaminated food and water; however, the role of healthcare in exposing individuals to EDCs is grossly underappreciated. Indeed, through the use of medications as well as medical equipment and devices, healthcare providers are unknowing mediators of exposure to EDCs, chemicals that might not only promote disease but that may also antagonize the efficacy of treatments. The ethical implications of provider-dependent exposure are profound. A failure to disclose the endocrine-disrupting properties of medical interventions violates core principles of nonmaleficence, patient autonomy, and justice as well as the practice of informed consent. Furthermore, physicians’ lack of knowledge regarding EDCs in medical practice artificially skews risk–benefit calculations that are fundamental to informed medical decision-making. To combat this underappreciated ethical challenge, urgent action is required. Healthcare providers must be educated about endocrine disruption. Known EDCs, defined by endocrinologists, should be clearly labeled on all medical products, and all medication components and devices should be screened for endocrine-disrupting properties. Finally, communication strategies must be devised to empower patients with knowledge about these risks. Providing ethically competent care requires an open acknowledgment of endocrine risks imposed by the medical community that have heretofore been ignored.

“A good intention, with a bad approach, often leads to a poor result.”

—Thomas Edison

The provision of medical care puts patients into direct contact with endocrine-disrupting chemicals (EDCs), unbeknownst to them and their providers. This raises critical ethical questions that must be addressed by the medical community. Defined as “an exogenous chemical, or mixture of chemicals, that interferes with any aspect of hormone action” (1), scientific evidence accumulated over the last 25 years now implicates EDCs in the pathogenesis of various reproductive, neuropsychiatric, and metabolic disorders (1). Moreover, exposures occurring during sensitive developmental windows are now thought to confer long-term disease risk (1). While it is widely acknowledged that EDC exposures occur through use of personal care and home products, environmental contamination arising from industrial activity and agriculture, as well as contaminated food and water, less appreciated is the role of medical care as an exposure source. Indeed, it is clear that medications and medical supplies contain EDCs, and some of our most vulnerable patients are those most highly exposed. It is time for a reckoning in the healthcare community to address our role in exposing patients to potentially harmful chemicals and to devise a path forward to address the ethical and clinical implications of this poorly understood iatrogenic risk.

## Endocrine Disruptors in Medications

While some active ingredients in medications are designed to modulate hormonal activity, largely unrecognized is the potential for “inactive” ingredients to exert hormone-disrupting properties. Defined by the Food and Drug Administration (FDA) as “any component other than the active ingredient in a drug product,” the list of inactive ingredients in formulations of medications include classes with known endocrine-disrupting properties. For example, phthalates are a class of nonpersistent EDCs linked to reproductive and metabolic disorders in cell-based, animal, and epidemiological studies (1). Phthalates are incorporated into medications to control release in the gastrointestinal tract for more precise drug delivery (2). Indeed, many drugs used to treat gastrointestinal disorders contain phthalates, including 5-aminosalicylates, protein pump inhibitors, bisacodyl, probiotic supplements, and pancreatic enzymes (2). Moreover, use of these medications is associated with increased urinary phthalate levels. In National Health and Nutrition Examination Survey (NHANES) data, urinary phthalate levels among mesalamine users were 50 times higher than nonusers (3). Importantly, exposures are not restricted to medications used in gastroenterology. An analysis of prescription and over-the-counter medications in the United States and Canada identified 6 prescriptions containing dibutyl-phthalate (DBP) and 45 prescriptions containing diethyl-phthalate (DEP); among over-the-counter medications, 3 agents contained DBP and 64 contained DEP (4). Among medications sold in Denmark, 154 drugs were found to contain 5 different phthalates, with 5 medications exceeding the recommended limit of DBP: mesalamine, multienzyme, budesonide, lithium, and bisacodyl (5). In NHANES data, elevated urinary phthalates were associated with use of omeprazole, theophylline, and didanosine (3). In a separate Danish study, DBP exposure exceeded recommended levels in 50% to 90% of patients by as much as 6-fold, with the greatest impact coming from lithium (6). Importantly, evidence suggests that medication-associated DBP exposure disrupts hormonal axes. In a crossover-crossback study of patients treated with mesalamine for inflammatory bowel disease, conversion to mesalamine products containing DBP altered levels of thyroid hormones and antithyroid antibodies (7). Parabens are an emerging class of EDCs that disrupt multiple signaling pathways and hormone-dependent outcomes, including those related to sex steroids, glucocorticoids, and thyroid hormones, among others (1). Parabens are employed as inactive ingredients for their antimicrobial properties and are exempt from tolerance requirements because they are “generally recognized as safe” by the FDA (8). In an analysis of patients taking medications potentially containing parabens (e.g., fluoxetine, ibuprofen, diphenhydramine, acetaminophen, and dextromethorphan/guaifenesin), use of these medications was associated with elevated urinary levels of propylparaben and nearly significant elevations in methylparaben. While the overall results were driven by 1 patient who took the medication shortly before sampling, the authors concluded that medications may contribute to high paraben exposures (8). In NHANES, urinary ethylparaben levels were higher among alendronate users than nonusers; specifically, 3 of 76 patients taking alendronate had very high urinary ethylparaben levels (9). Furthermore, 18% of alendronate users were found to have urinary butylparaben levels above the 95th percentile (9). Taken together, these data indicate that medications are an underappreciated source of exposure to multiple EDC classes. Importantly, this evidence underrepresents total medication-associated EDC exposure since studies have been limited to a subset of medications and only a few known EDCs.

## Endocrine Disruptors in Medical Equipment

Medical supplies are another significant source of exposure to several important classes of EDCs, including phthalates, bisphenol A (BPA), parabens, perfluoroalkyl substances, and triclosan. In an analysis of common medical supplies, DEP, butylparaben, and BPA were released by syringes; microcapillary blood tubes released methylparaben, ethylparaben, and propylparaben; and venous catheters released methylparaben, ethylparaben, propylparaben, butylparaben, bisphenol S, DBP, and DEP (10). Perhaps of even greater concern is evidence of release into parenterally administered solutions, including multiple parabens, phthalates, and other putative EDCs (10).

The presence and release of phthalates is unsurprising since they account for up to 30% to 40% of the weight of medical-use plastics into which they are noncovalently bound (11,12). Importantly, lipophilic solutions such as parenteral nutrition and blood enhance phthalate leaching (13). Among the critically ill, the diversity of potential exposure sources is striking. Endotracheal tubes, blood bags, and cardiopulmonary bypass machines as well as intravenous bags, extension lines, and infusion sets all may contain phthalates (12-15). Even after France significantly restricted phthalate use in medical supplies, hospital exposures persisted from intravenous extension lines, blood transfusion sets, and extracorporeal membrane oxygenation machines (14). Indeed, in a study of hospitalized pregnant women in France, 74% were found to be exposed at least once to a phthalate-containing medical device (11).

Parabens are often used in medical supplies for their broad-spectrum antimicrobial activity. Their concentrations in intravenous solutions can be as high as 0.72% (for methylparaben) (10). Parabens are also incorporated into ultrasound gels for their bacteriostatic activity (16). Despite their proposed antimicrobial function, one Canadian study concluded that parabens are only marginally effective at inhibiting bacterial growth and “should not be viewed as a protective measure in preventing bacterial contamination” of ultrasound gel (16). Some hospitals also use parabens in heparin lock solutions to prevent catheter-related bloodstream infections (17). In an intriguing analysis at St. Jude Children’s Research Hospital, removal of parabens from heparin lock solutions was associated with an increase in catheter-related bloodstream infections, prompting a decision to restore parabens to heparin locks for all pediatric cancer patients (17). In this instance, the benefits of parabens may be significant; however, it remains unclear how those advantages are counterbalanced by endocrine risks and whether agents that do not modulate hormone activity would offer similar benefits with less hormonal uncertainty.

BPA is a ubiquitous EDC that modulates multiple endocrine and metabolic signaling pathways and is linked to reproductive, neuropsychiatric, and metabolic disorders through a diversity of laboratory- and population-based studies (1). In a clinical context, BPA exposure likely occurs most commonly during dialysis. Dialyzers leach BPA even after rinsing with normal saline, and since hemodialysis can expose patients to more than 300 L of water weekly, potential exposure to BPA is substantial (18). In addition to dialysis, BPA-based resins are used to coat aluminum ointment tubes (19). In tubes with high amounts of BPA-based resin, over 90% of the extractable BPA migrates into the ointments during storage (19). This is significant because BPA is absorbed transdermally. In sum, the available evidence clearly demonstrates that medical devices are potential sources of EDC exposure; however, as with medications, this risk is likely understated since our knowledge is restricted to a few known classes of EDCs and a limited set of medical devices.

## The Neonatal Intensive Care Unit as a Special Case

Based on expansive evidence that EDC exposures during sensitive developmental windows exert deleterious effects on long-term health, neonatal intensive care unit (NICU)-associated exposures are likely important. Phthalate exposures in the NICU were first reported in 2004 (20), where it has been suggested that exposure levels markedly exceed those “estimated” to be safe to avoid adverse toxicity (21). Importantly, these early life exposures may exert adverse effects on health. Among NICU patients, phthalates are associated with broncho-pulmonary dysplasia, necrotizing enterocolitis, parenteral nutrition-associated cholestasis, and neurodevelopmental disorders (22). While the direction of causality may be complex, potential exposure-induced adverse effects warrant careful investigation, especially given evidence that switching from phthalate-containing medical devices may reduce patient risk, as shown for cholestasis associated with total parenteral nutrition (23).

Importantly, NICU-associated EDC exposures are not restricted to phthalates. Urinary BPA levels among NICU patients are associated with the number of medical devices used in patient care (24). Urinary BPA levels were also elevated in NICU babies exposed to phthalate-containing products, illustrating the challenge of combinatorial EDC exposures in the critical care setting (25). In a recent analysis of 52 common NICU items, the authors found that three-fifths of equipment contained BPA and fourth-fifths contained parabens (26). Critically, this study also demonstrated that extracts from the medical supplies altered endocrine activity in cell lines, with 25% of the extracts demonstrating estrogenic activity and 10% showing anti-androgenic activity (26). Given the critical organizational role of hormones during development, these endocrine-disrupting activities raise concerns about long-term health risks.

## Medical Ethics of Endocrine Disruptors in Clinical Care

As evidence emerges demonstrating that medications and medical equipment contain EDCs, several questions arise for the practicing clinician. First, what are healthcare providers’ ethical obligations to disclose potential adverse endocrine effects of “inactive” ingredients in the medications they prescribe or medical equipment they use? Second, is informed consent dependent upon disclosing all “inactive” ingredients to patients? Finally, what steps should be taken by the medical profession, the FDA, and other regulatory bodies to identify EDCs used in clinical care, quantify the clinical impact of those exposures, and restrict or ban ingredients with known risks? To date, these issues remain woefully unaddressed despite clear ethical mandates.

The ethical obligation of nonmaleficence requires that healthcare providers “do no harm” to their patients; this includes providing patients with important information necessary for making decisions about their care. Thus, prescribers should disclose all needed information related to potential side effects, which should include the impact of “inactive” ingredients if such agents have potential to exert harm. Should such an adverse effect be possible, the provider is further obligated to diligently conduct follow-up assessments and intervene when needed. Perhaps a more overriding obligation related to harm would be that medical, scientific, and regulatory entities further study the use of specific ingredients to develop new formulations without endocrine-disrupting properties. However, where the evidence is already robust, the ethical mandate is for action from clinicians, manufacturers, and regulatory agencies to protect patient health.

Built upon principles of justice and autonomy, informed consent requires that decision-makers be assessed for their capacity to freely make decisions, be given the relevant information needed to make an informed decision, verbalize understanding about the possible options related to a specific decision, and provide an independent and voluntary decision. Information must include risks, expected benefits, and expected side effects. In addition, this information must be provided in a language and at an academic level that the person making the decision can understand. Thus, there is an inherent obligation of providers to know all potential care-associated EDC exposures and their potential adverse effects on the patient. Critically, although every ingredient used in a drug must be disclosed according to the US Pharmacopeia, many consumer prescription medications packaged in a pharmacy vial lack an accompanying ingredient label. Knowledge of chemicals used in medical devices is even less common. Therefore, such information must come from the provider who has, or should have, access to full label information.

In clinical practice it is important to look at the equipoise, or balance, of harm versus utility of any additive or ingredient in all medications. With all medical interventions, there is a risk–benefit analysis related to the promotion of health and the avoidance of harm. If there is enough evidence that the harm of a medication does not justify its use, it should not be used. Where benefits may still outweigh risks, careful review of the full ingredient list and identification of alternatives without harmful adjuvants may empower the provider to choose safer alternatives. Indeed, in an analysis of Cardizem CD (branded diltiazem), 1 of the 5 available strengths listed phthalates in its formulation, whereas the other 4 strengths did not; this was in contrast to generic diltiazem formulations that all contained phthalates (4). Among medical supplies, it is interesting to note that 1 butterfly blood draw kit released significantly higher amounts of parabens than other kits (10). Collectively, this suggests that safer options are possible. Importantly, clinical decision-making is complicated by polypharmacy, as this increases the likelihood and burden of exposure to potential endocrine-disrupting adjuvants, while also implying that the exposure will occur in an individual with impaired physiological reserve due to pre-existing comorbidities. The risk–benefit calculation for both patients and providers is markedly more difficult in such scenarios, but it is still essential for clinicians to advise the patient of risk in these situations.

## The Challenges and a Path Forward

With clear evidence of patient exposure to EDCs during the course of clinical care coupled with an ethical mandate upon healthcare providers to minimize risk, it is incumbent upon the medical community to ask why this issue has not been addressed and to identify a just path forward. A central challenge is that chemicals used in the United States and, until recently, in Europe are not regulated based on their endocrine-disrupting potential; moreover, screening of chemicals for endocrine activity is woefully inadequate. Thus, the previous discussion is inherently restricted to the small subset of *known* EDCs analyzed in a *limited subset* of medications and medical supplies; the potential for unknown additional exposures of endocrine significance is both real and concerning. Furthermore, there are significant knowledge deficits among providers. This includes a lack of understanding of endocrine disruption as well as a dearth of knowledge regarding the chemical content and potential exposures arising from medication adjuvants and medical equipment. In short, clinicians cannot meet their ethical obligations of informed consent if risks remain hidden.

**Figure 1.**
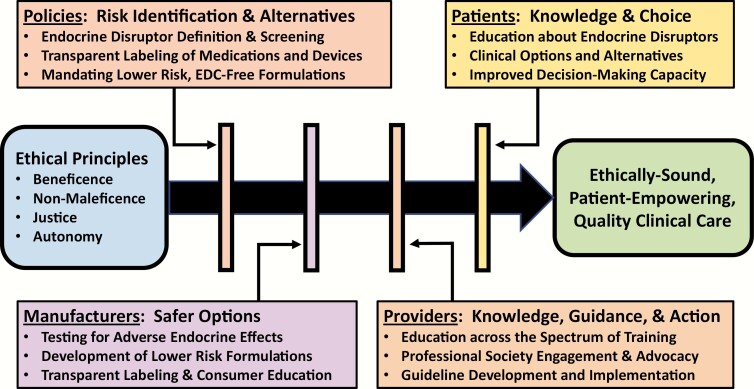
Ethical care in the context of healthcare-related exposure to endocrine-disrupting chemicals. Applying the core principles of medical ethics to clinical care in the context of healthcare-related EDC exposures requires changes on the part of multiple stakeholders, including policymakers, industry, providers, and patients.

Thus, the path forward is one paved with information and transparency (Fig. 1). The full chemical content of medications and medical equipment must be freely and easily accessible to both patients and providers. The potential endocrine toxicity of those chemicals must be assessed both individually as well as in the common combinations used in medical formulations. Furthermore, safety should be proven and not assumed, and safer alternatives must be developed. If manufacturers must use EDCs in their products, they should be required to provide a rationale, as is currently required in Europe for the use of phthalates in medical supplies intended for neonates and pregnant women (12). Finally, healthcare providers must be educated about the science of endocrine disruption. Central to these efforts must be the active engagement of the endocrine community in defining and identifying EDCs based upon sound science rooted in the promotion of health and resistant to manufactured doubt. Given the centrality of endocrine signaling to normal development and physiology, alterations in hormone signaling by EDCs in medications and medical devices may modulate the beneficial effects of medications and medical interventions across diverse disease states. Knowledge of these effects may empower new formulations that amplify therapeutic efficacy. In the end, this knowledge is likely to reduce risk and improve patient care.

## Data Availability

Data sharing is not applicable to this article as no datasets were generated or analyzed during the current study.

## Additional Information

***Disclosure Summary:*** RMS declares that he has received honoraria from CVS/Health and the American Medical Forum.
